# Knowledge About HIV/AIDS and Its Transmission and Misconception Among Women in Bangladesh

**DOI:** 10.34172/ijhpm.2022.6321

**Published:** 2022-02-08

**Authors:** Jahar Bhowmik, Raaj Kishore Biswas

**Affiliations:** ^1^Department of Health Sciences and Biostatistics, Swinburne University of Technology, Hawthorn, VIC, Australia.; ^2^Transport and Road Safety (TARS) Research Centre, School of Aviation, University of New South Wales, Sydney, NSW, Australia.

**Keywords:** HIV/AIDS knowledge, Awareness, Women, Bangladesh, Spatial Distribution

## Abstract

**Background:** Asian and pacific region countries are high risk countries for human immunodeficiency virus/ acquired immune deficiency syndrome (HIV/AIDS). Although the prevalence of HIV/AIDS is low in Bangladesh but women in Bangladesh have been identified as susceptible due to associated socioeconomic exposures. There are various misconceptions associated with HIV/AIDS transmission among the women in low- and middle-income countries including Bangladesh, which lead to a negative attitude towards the HIV/AIDS-infected. The purpose of this study was to assess the overall knowledge, transmission, and misconception about HIV/AIDS among the women in Bangladesh as well as its spatial distribution across the country.

**Methods:** The study used data from the UNICEF (United Nations Children’s Fund) Multiple Indicator Cluster Survey (MICS) 2019, with a sample of 64 346 women. This was a cross-sectional, population-based survey of Bangladeshi women aged 15–49 conducted using a multistage, cluster sampling technique. Three binary outcome variables considered were knowledge about HIV/AIDS, knowledge about HIV/AIDS transmission and knowledge on myths and misconceptions on HIV/AIDS along with 10 predictors based on past literature. Bivariable analysis using chi-square tests of association was conducted to examine the unadjusted percentage differences of the outcome variables for each of the predictor variables and their associations. Multiple binary logistic regression models were then fitted to evaluate the association between the outcome variables and the predictors after adjusting for survey cluster, strata, and weights. All analysis was conducted in R software (V 2.5.0).

**Results:** The percentage of women who held knowledge about HIV/AIDS, knowledge about HIV/AIDS transmission and knowledge on misconceptions about transmission of HIV were on average 60.3%, 52.2% and 71.7% respectively. The models indicated that women regularly exposed to media were 79%, 18% and 19% significantly more likely (odds ratio, OR: [95% CI] = 1.79: [1.70, 1.89]; 1.18: [1.10 1.26] and 1.19: [1.11, 1.27]) to have heard about HIV, aware about HIV transmission, and have less misconceptions about HIV respectively compared to those who were not exposed to media. Overall results indicate that women from peripheral districts living far from metropolitan cities were most unaware of HIV and had higher misconceptions about AIDS.

**Conclusion:** The findings of this study should assist policy-makers and program implementers to focus on raising awareness to educating women about how HIV/AIDS is transmitted. Furthermore, interventions should be made by targeting the most disadvantaged groups, including younger women with low education living in rural areas, from poor households and limited access to information. Also, education on HIV transmission in Bangladesh should integrate cultural and ethnic considerations of HIV/AIDS.

## Background

**Key Messages**

** Implications for policy makers**

- Literature lacks a detailed assessment on human immunodeficiency virus/acquired immune deficiency syndrome (HIV/AIDS) awareness on Bangladesh.
- Women from peripheral districts living further away from divisional cities had more misconceptions.
- Media and mobile phones could be an avenue for disseminating awareness in Bangladesh.

** Implications for the public**

It is important to publicize knowledge, transmission process and various misconceptions of sexually transmitted diseases (STDs) to increase public health awareness. When it comes to public health and acquired immune deficiency syndrome (AIDS), awareness about human immunodeficiency virus (HIV) can make a major difference. Sharing correct information about HIV/AIDS with each other is important in reducing the spread of these deadly diseases. Findings from this study show that regular access to media and keeping up to date information via telecommunication can help aware women regarding HIV/AIDS. Greater focus required for most vulnerable groups including women of younger ages, particularly those with lower education living in rural areas and from poor households who do not have access to accurate information. Community engagement through the awareness campaign leading by the community leaders could increase the knowledge about HIV and its transmission which eventually could minimize the misconception.

Human immunodeficiency virus (HIV), the virus that causes acquired immune deficiency syndrome (AIDS) has become one of the world’s most serious public health challenges in recent years. According to the Joint United Nations Programme on HIV/AIDS (UNAIDS),^1^ there were 37.9 million people globally living with HIV in 2018, which is 3.3% higher than 2015. Asia and the Pacific region has the second highest prevalence of HIV in the world, with an estimated 5.1 million people in 2015^2^ and Bangladesh is part of this region with a relatively low prevalence of HIV.^3,4^

One of the UNAIDS targets to eliminate AIDS was to reduce the number of newly infected HIV cases to less than 500 000 globally by 2020, similar to the 90-90-90 target.^5^ The indicator to achieve this target was to ensure that 90% of young people have the skills, knowledge, awareness and capacity to protect themselves from HIV and have access to sexual and reproductive health services by 2020 in order to reduce the number of new HIV infections among young women under 100 000 per year.^6^

Despite the low prevalence of HIV/AIDS, Bangladesh faces a high risk of fast spread of HIV/AIDS due to rapid urbanisation, rising unemployment, economic problems, poor medical facilities, lack of sufficient screening practices, lack of women’s autonomy, and unsafe sexual practices.^7-9^ Although Bangladesh has a National AIDS Committee since 1985 and National Strategic Plan 2004-2010 addressed five priority areas for HIV interventions,^10^ the prevalence of HIV across the country is not homogeneous and operationalizing the policies require more data and assessment on people’s perception on AIDS and its transmission.

According to the Global AIDS Monitoring,^6^ young people with comprehensive knowledge of HIV prevention and transmission are defined as (1) knowing that consistent use of a condom during sexual intercourse and having one uninfected faithful partner can reduce the chance of getting HIV, (2) knowing that a healthy-looking person can have HIV, and (3) rejecting the two most common local misconceptions about transmission/prevention of HIV. HIV/AIDS related knowledge and education are seen by many as central to increasing people’s awareness of, as well as decreasing their vulnerability to HIV/AIDS.^8,11-13^ Prevalence of HIV/AIDS is found to be significantly higher among people who are unaware of the potential routes of transmission,^14,15^ which infers that HIV/AIDS related knowledge and education helps in decreasing their vulnerability to HIV/AIDS.

A national survey found that only 38% of people surveyed in Bangladesh could identify two or more routes of HIV transmission, and only 40% could recognize two or more methods of prevention.^16^ Several potential factors that attributable to the increased risk of HIV infection and/or transmission are education, poverty, place of residence (urban/rural), gender inequity, migration, knowledge about HIV/AIDS, ethicality and exposure to media.^7,8,17,18^ In Bangladesh, the level of awareness and knowledge about the correct ways to avoid HIV/AIDS transmission among the women are relatively low. Hossain et al^9^ reported that the level of education of women in Bangladesh is significantly associated with their knowledge and awareness concerning sexually transmitted diseases (STDs).

The lowest awareness about HIV/AIDS transmission was found among rural uneducated women which was only 20% in Bangladesh.^16^ The importance of mass media for health promotion and disease prevention is well known.^19^ Asaduzzaman et al revealed a significant association between exposure to mass media and awareness of HIV/AIDS among the ever-married women in rural Bangladesh.^20^ Many studies recommended to use mass media as a tool to engage wider audience in creating awareness and misconception regarding HIV/AIDS especially for the women living in regional and remote areas.^7,9,18^

Several studies have addressed the knowledge and attitudes about HIV/AIDS among women in Bangladesh and its associated sociodemographic factors. However, there is a lack of recent large-scale population-based research on knowledge about HIV/AIDS, knowledge about HIV/AIDS transmission and knowledge about misconception of HIV/AIDS among the women in Bangladesh, and possible actionable factors which can be used for effective interventions to assist in achieving Sustainable Development Goal 3 by 2030.^21^ Identifying the vulnerable cohorts at risk of HIV is crucial to efforts to address HIV, in regard to prevention, treatment or care and support. Thus, the objectives of this study were to conduct a district-wise spatial analysis on the population proportion of women regarding their knowledge on HIV/AIDS, and misconceptions about HIV/AIDS, and to assess their association with the possible risk factors.

###  Theoretical Framework

The conceptual framework of current study was framed on the health belief model (HBM), which suggests that an individual’s course of action is determined by their understanding of the perceived threat of a disease, and benefits of action against the disease.^22,23^ In the framework of the HBM, it is hypothesized that to implement recommended health behaviour, the individual must be familiar with the possible negative outcomes, for instance, to have heard about HIV/AIDS transmissions and its prevention methods.^23-25^ This model has been used in past studies on HIV/AIDS prevention to address HIV/AIDS knowledge, methods of transmission, and its myths and facts.^26-29^ This study aims to explore how the perception of women about HIV/AIDS in Bangladesh can be affected by level of knowledge on HIV/AIDS, socio-demographic factors, exposure to mass media, ownership of mobile phone, and available interventions and/or their barriers by evaluating Multiple Indicator Cluster Survey (MICS) 2019 data. Understanding the vulnerable communities who are more likely to be oblivious to the risks of HIV would help set interventions and further studies on the behaviours on these cohort. Therefore, considering these objectives, the theoretical framework of the HBM^30^ was adopted in this study.

## Methods

###  Sources of Data

This study used the secondary data of the latest sixth round Bangladesh MICS conducted in 2019. This is a cross-sectional, population-based survey of Bangladeshi women aged 15–49 conducted from January 19 to June 1, 2019 using a multistage, cluster sampling technique.^31^ As part of the Global MICS Programme, this survey was carried out jointly by the Bangladesh Bureau of Statistics and the United Nations Children’s Fund (UNICEF) Bangladesh. The estimation of the sample size and sampling method has been designed to provide estimates for a range of public health indicators on children and women at the national level for eight divisions and 64 districts.^31^

The sample of households were selected in two stages, sampling was performed by region and then stratified for area type (urban or rural). From 64 districts (stratum), a number of primary sampling units (PSUs), consider as clusters, were selected using probability proportional to size sampling procedure. Cluster numbers per region were calculated according to sample size estimations, and each cluster (PSU) included 20 households. Within each cluster, the random systematic selection was used to select 20 households. After a household listing was carried out within the selected enumeration areas, a systematic sample of 20 households was drawn in each sampled PSUs. The number of PSU and number of sampled households in the survey were 3220 and 64 400 respectively. A more detailed description of the sampling design, all the questionnaires of MICS and the data that supports the findings of this study are available from https://mics.unicef.org/tools?round=mics6.

###  Measures

####  Outcome Variables

Based on prior literature, we have used three outcome variables.^23,32-34^ The outcome variables were knowledge about HIV/AIDS, knowledge about HIV/AIDS transmission and knowledge about misconception of HIV. First outcome variable awareness on HIV/AIDS was assessed with the question: ‘Have you ever heard of HIV or AIDS?’ It was scored dichotomously (‘yes’ = 1 and ‘no’ = 0).^23^ The second outcome variable was knowledge about HIV/AIDS transmission. It was a score measure created from two questions asking whether women knew that they can protect themselves from getting HIV/AIDS by getting response on (*i*) ‘Can people reduce their chance of getting HIV by having just one uninfected sex partner who has no other sex partners?’and (*ii*) ‘Can people reduce their chance of getting HIV by using a condom every time they have sex?’ Respondents who correctly answered both questions were coded as ‘yes’ = 1, otherwise as ‘no’ = 0 regarding their knowledge on HIV/AIDS transmission.^23^ The third outcome variable was also a binary score measure quantifying knowledge on myths and misconceptions on HIV/AIDS based on the responses to four relevant survey questions. The fours questions were: (*i*) ‘Can people get HIV from mosquito bites?,’ (*ii*) ‘Can people get HIV by sharing food with a person who has HIV?,’ (*iii*) ‘Can people get HIV because of witchcraft or other supernatural means?,’ and (*iv*) ‘Is it possible for a healthy-looking person to have HIV?’ Answers to all four questions were combined and recorded into binary variable by coding ‘yes’ = 1 if a respondent could correctly response to at least two of the four questions and ‘no’ = 0 otherwise.^23,34^ The sample size for all three outcomes varied as not every respondent answered all queries; for example, those unaware of HIV or AIDS were not asked any follow up questions regarding transmission or misconceptions.

####  Independent Variables

According to past literature, parameters of the HBM, and pre-analysis results, ten predictors were included in this study as explanatory variables.^7,13,17,23^ The selected explanatory variables are age of respondents (≤25, >25); area of residence (urban, rural); education of both respondent and her partner (none/pre-primary, primary, secondary, higher secondary and above); wealth index (poorest, poorest, middle, richer, richest); gender of house head (male, female); age of house head (15-34, 35-50, 50+); division (Dhaka, Barishal, Sylhet, Khulna, Rajshahi, Chattogram, Rangpur, Mymensingh); media exposure (yes, no) and mobile phone ownership (yes, no). For media exposure, if the respondent said yes to watching tv or listening to radio or reading newspaper or used internet in past week, she was considered as exposed to media (yes). The inbuilt household wealth index was based on asset variables compiled using principal component analysis.^31^ Survey weights, strata and cluster information were also extracted for model adjustment.

###  Statistical Analysis

 Descriptive analysis was used to estimate the percentage of HIV/AIDS knowledge, prevention and transmission knowledge among women. Initially a bivariable analysis was conducted where the percentage of HIV/AIDS knowledge, knowledge on transmission of HIV/AIDS, and knowledge on the misconception of HIV/AIDS among women according to various factors including age group, marital status, place of residence, ethnicity, educational attainment, and household socioeconomic status were also calculated. These associations were tested using Chi-square tests. Following that generalised linear models with binary outcomes adjusting for survey cluster, strata and weights were conducted, which provided greater scope of generalization and adjusted effect sizes. All three binary logistic regression models fitted for three outcome variables were controlled for all the sociodemographic factors to address possible confounding effects.

 The HIV/AIDS knowledge, knowledge on transmission and knowledge on misconception among women were mapped among the 64 rural and urban districts in Bangladesh using spatial graphs for comparison. The modelling and mapping were conducted using R-package ‘survey’ and ‘maps’ and ‘ggplot2.’ All data compilations, and analyses were conducted in R (3.5.0).

## Results

 In the MICS 2019, data from 64 346 women aged between 15 and 49 years were available for analysis. However, sample size varied for each outcome depending on missing responses. The mean age of respondents was 30.01 (standard deviation=9.67) years. About 37.4% (N = 23 832) of the women were aged below 26 and rest were aged 26 or more. Around 16.1% of the women had no or pre-primary education, 14.7% had primary, 44.8% had secondary, and 15.9% had higher secondary or above education. Most of the women (80.1%, N = 51 019) resided in rural areas.

Majority (N = 38 394, 60.3 %) of the women reported that they heard about HIV/AIDS (Table 1). 67.2% of them knew that having one uninfected sex partner reduces risk of getting HIV/AIDS and 61% were aware that using condoms could help prevent HIV/AIDS. Most of the respondents (95.8 %) knew that HIV could not be caused by supernatural means which is the highest correct answer percentage among the four questions on myths and misconceptions. The lowest correct response was for the question on whether a healthy-looking person can have HIV (58.8%).

**Table 1:** Percentage of Correct Answers by Questions Among Women Who Reported Knowledge, Knowledge of Transmission and Knowledge of Misconception About HIV/AIDS

**Questions**	**N**	**%**
Heard of HIV/AIDS	38 394	60.3
Can get HIV through supernatural means?	36 745	95.8
Can get HIV from mosquito bites?	26 093	68.1
Can avoid HIV by having one uninfected partner?	25 725	67.2
Can avoid HIV by using a condom correctly every time?	23 207	60.6
Can get HIV by sharing food with a person who has HIV?	23 674	61.7
Healthy-looking person may have HIV?	22 354	58.3

Abbreviation: HIV/AIDS, human immunodeficiency virus/acquired immune deficiency syndrome.

**Figure 1:**
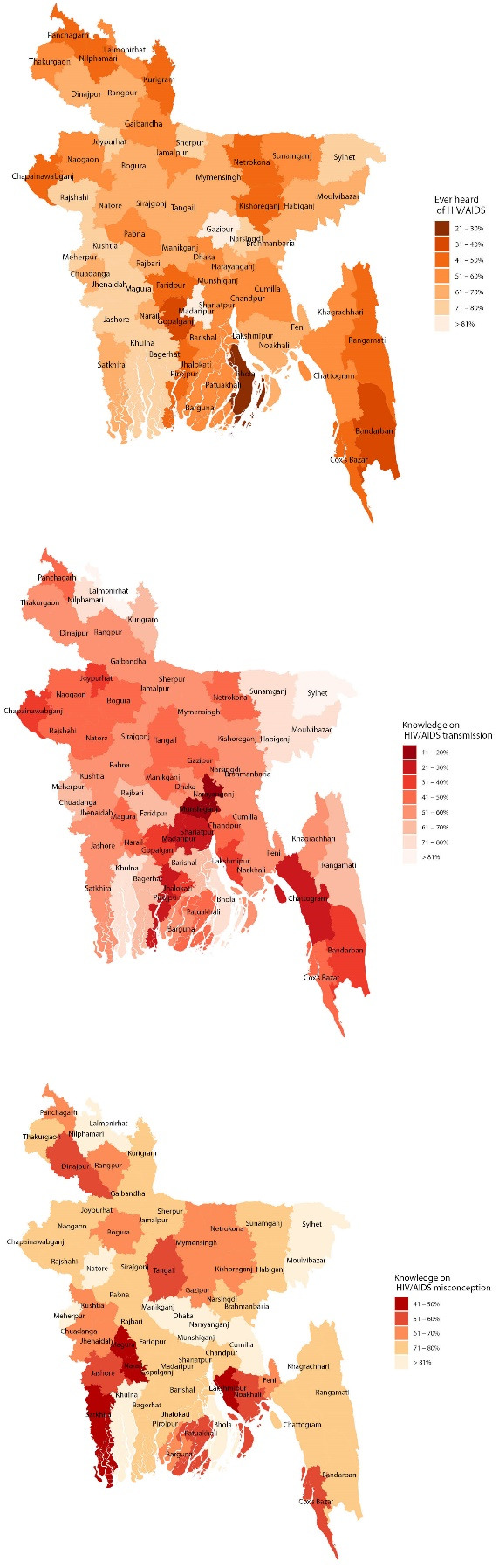
Geospatial Distribution of Women’s Knowledge About HIV/AIDS, Its Transmission and Various Misconceptions Among 64 Districts in Bangladesh. Abbreviation: HIV/AIDS, human immunodeficiency virus/acquired immune deficiency syndrome.

In the survey, knowledge about HIV/AIDS, knowledge about HIV/AIDS transmission and misconceptions about transmission of HIV among the women were 60.3% (N = 38 394), 52.2% (N = 19 964) and 71.7% (N = 27 526) respectively. These percentages varied among the major districts according to their geographical location and socioeconomic status. The highest percentage of knowledge, knowledge of transmission and misconceptions about HIV/AIDS among women were 84.1% in Madaripur, 88.5% in Sylhet and 89.8% in Dhaka respectively and the lowest were 22.9% in Bhola, 19.4% in Narayanganj and 40.3% in Narail respectively. Figure 1 show the mapping of women’s knowledge about HIV/AIDS, knowledge about HIV/AIDS transmission and misconceptions about transmission of HIV/AIDS among 64 districts in Bangladesh which shows that the percentages were lower in the regional and remote part of the country, especially in Bhola, Bandarban, Satkhira, narial, Gopalganj, Panchagarh, and overall higher in the urban districts of the country. However, some districts in the urban areas including Tangail and Chattogram also reported lower knowledge about HIV/AIDS and knowledge about HIV/AIDS transmission (Figure 1). Overall, the percentage of women’s knowledge about HIV/AIDS, knowledge about HIV/AIDS transmission and knowledge of misconception about HIV/AIDS are higher in the regional and peripheral districts compared to central metropolitan areas.

**Figure 2:**
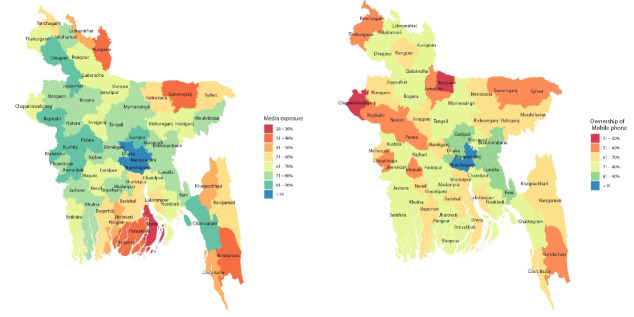
Geospatial Distribution of Women’s Exposure to Media and Ownership of Mobile Phone Among 64 Districts in Bangladesh.

The distributing of exposure to media and ownership of mobile phone, and their mapping among 64 districts were presented in Figure 2. The percentage of women exposed to media was highest in Dhaka district (94.3%) and lowest in Bhola (28.7%). The highest and lowest proportion of ownership of mobiles phones were in Narayanganj (93.2%) and Sherpur (46.6%) respectively. Overall, this percentage of exposure to media and ownership of mobile phone were higher in the urban districts and lower in the regional and remote districts. As can be seen from Figure 2, the vulnerable cohort of women were those who were living in the remote and regional districts, for example Bhola, Kurigram, Borgona, Bandarban and Sunamganj had the lowest exposure to media (<40%). The results presented in Figures 1 and 2 show that there is an association between exposure to media and overall knowledge about HIV/AIDS among the women in Bangladesh, which suggests that the rural and remote areas had less exposure to media and residents were less aware about HIV/AIDS and its consequences. Similarly, results presented in Figures 1 and 2 indicate that respondents form the rural and remote areas had less (<50%) or no ownership of mobile phone and were less aware about HIV/AIDS and its consequences.

The bivariable association between the three outcome variables and potential sociodemographic factors are presented in Table 2. All sociodemographic factors were significantly associated with knowledge about HIV/AIDS (*P* < .001). Apart from age, knowledge of HIV/AIDS transmission was significantly associated with rest of the sociodemographic factors including education of women, education of house head, wealth index, area of residence, division, age and gender of house head, exposure to media and ownership of mobile phone (*P* < .05). Similarly, apart from age of house head, all sociodemographic factors were significantly associated with knowledge on HIV/AIDS misconceptions (*P* < .001).

**Table 2:** Distribution of Knowledge, Transmission, and Misconceptions of HIV/AIDS Across Selected Sociodemographic Factors

**Variables**	**Heard of HIV/AIDS ** **(N = 63 659)**		**Knowledge on HIV/AIDS ** **Transmission (N = 38 281)**		** Knowledge on Misconception About HIV/AIDS (N = 38 368)**	
	**Yes (%)**	***P*** ** Value**	**Yes (%)**	***P*** ** Value**	**Yes (%)**	***P*** ** Value**
Age						
≤25	16 999 (71.3)	<.001	8817 (52.1)	.996	12 333 (72.6)	<.001
>25	21 395 (53.7)		11 147 (52.2)		15 193 (71.0)	
Education						
None or pre-primary	2330 (22.6)	<.001	875 (37.6)	<.001	1492 (64.1)	<.001
Primary	5935 (40.3)		2453 (41.5)		3919 (66.2)	
Secondary	20 433 (71.6)		10 226 (50.2)		14 355 (70.3)	
Higher secondary	9706 (95.8)		6410 (66.3)		7760 (80.0)	
Wealth index						
Poorest	4947 (37.7)	<.001	2148 (43.5)	<.001	3126 (63.3)	<.001
Poorer	6720 (50.8)		3167 (47.3)		4454 (66.3)	
Middle	9381 (72.3)		4204 (50.3)		5770 (68.9)	
Richer	9381 (72.3)		5036 (53.9)		6867 (73.2)	
Richest	8967 (82.0)		5409 (60.5)		7309 (81.6)	
Area of residence						
Rural	29 326 (57.5)	<.001	14 899 (51.0)	<.001	20 526 (70.0)	<.001
Urban	9068 (71.7)		5065 (56.0)		7000 (77.2)	
Division						
Dhaka	7916 (62.1)	<.001	3502 (44.3)	<.001	6135 (77.5)	<.001
Barishal	2657 (48.6)		1224 (46.2)		1899 (71.5)	
Chattogram	6651 (55.7)		3083 (46.6)		4630 (69.7)	
Khulna	7202 (71.8)		4005 (56.0)		4475 (62.2)	
Mymensingh	2010 (60.9)		1071 (53.5)		1327 (66.2)	
Rajshahi	4655 (62.1)		2061 (44.3)		3534 (76.0)	
Rangpur	4106 (52.9)		2506 (61.0)		2917 (71.0)	
Sylhet	3197 (65.2)		2512 (78.6)		2609 (81.6)	
Age of house head						
15-34	7598 (61.4)	<.001	3884 (51.3)	<.001	5395 (71.1)	.182
35-50	16 262 (59.1)		8340 (51.4)		11 642 (71.6)	
>50	14 534 (61.1)		7740 (53.4)		10 489 (72.2)	
Gender of house head						
Male	34 202 (60.0)	<.001	17 858 (52.4)	.018	24 424 (71.5)	<.001
Female	4192 (63.0)		2106 (50.4)		3102 (74.1)	
Education of house head						
None or pre-primary	9700 (45.3)	<.001	4534 (46.9)	<.001	6687 (69.0)	<.001
Primary	10 186 (56.9)		4952 (48.7)		6918 (68.0)	
Secondary	12 078 (70.9)		6431 (53.4)		8794 (72.9)	
Higher secondary	6430 (87.8)		4047 (63.1)		5127 (79.7)	
Media exposure						
No	8482 (41.2)	<.001	3927 (46.5)	<.001	5612 (66.2)	<.001
Yes	29 912 (59.4)		16 037 (53.8)		21 914 (79.6)	
Mobile phone ownership						
No	9409 (48.7)	<.001	4253 (45.5)	<.001	6246 (66.5)	<.001
Yes	28 985 (65.4)		15711 (54.3)		21 280 (73.5)	

Abbreviation: HIV/AIDS, human immunodeficiency virus/acquired immune deficiency syndrome.

 Among the women who had access to media, 59.4%, 53.8% and 79.6% of them had knowledge about HIV/AIDS, knowledge about HIV/AIDS transmission and knowledge about misconceptions of HIV/AIDS respectively. Among the eight divisions, knowledge about HIV/AIDS, knowledge about HIV/AIDS transmission and knowledge about misconceptions of HIV/AIDS transmission were highest in Khulna (71.8%), Sylhet (78.6%) and Khulna (37.8%) and the lowest were in Rangpur (52.9%), Dhaka & Rajshahi (44.3%) and Sylhet (18.4%) divisions respectively. The percentage of women in urban areas were reported 71.7%, 56% and 22.8% knowledge about HIV/AIDS, knowledge about HIV/AIDS transmission and misconceptions about transmission of HIV/AIDS respectively as compared to 57.5%, 51% and 30% respectively those who were from rural areas. Percentage of younger women (≤25) seemed to have higher knowledge about HIV/AIDS (71.3%) than older women (53.7%).

The fitted logistic regression models, where all variables were adjusted together for controlling confounding factors, found that all sociodemographic variables, apart from gender of house head, were associated with different levels of HIV knowledge after adjusting for survey weights, cluster and strata-wise variations (Table 3). For example, those with regular access to media were 79%, 18% and 19% significantly more likely (odds ratio, OR [95% CI] = 1.79 [1.70, 1.89]; 1.18 [1.10 1.26] and 1.19 [1.11, 1.27]; *P* < .001) to have had heard about HIV, aware about HIV transmission, and had less misconceptions about HIV respectively compared to those who did not have access to media. Similarly, women who owned mobile phone were 43%, 29%, and 12% more likely (OR [95% CI] = 1.43 [1.36, 1.51]; 1.29 [1.21 1.37] and 1.12 [1.05, 1.21]; *P* < .001) to have had knowledge about HIV/AIDS, aware about HIV transmission, and could correctly identify misconceptions about HIV than those who did not own mobile phone.

**Table 3:** Association Between Predictors and Knowledge, Knowledge of Transmission and Knowledge of Misconception About HIV/AIDS Among Women in Bangladesh: Results From Logistic Regression Models

**Variables**	**Heard of HIV/AIDS**		**Knowledge on HIV/AIDS Transmission **		**Knowledge on Misconception About HIV/AIDS**	
	**AOR (95 % CI)**	***P*** ** Value**	**AOR (95 % CI)**	***P*** ** Value**	**AOR (95 % CI)**	***P*** ** Value**
Age (ref: ≤25)						
>25	0.86 (0.81, 0.90)	<.001	1.23 (1.17, 1.31)	<.001	0.96 (0.91, 1.02)	.217
Education (ref: None or pre-primary)						
Primary	2.00 (1.87, 2.14)	<.001	1.18 (1.05, 1.33)	.006	1.12 (0.99, 1.25)	.068
Secondary	6.43 (5.99, 6.90)	<.001	1.79 (1.60, 2.00)	<.001	1.28 (1.14, 1.43)	<.001
Higher secondary	43.13 (37.54, 49.56)	<.001	3.35 (2.95, 3.79)	<.001	1.81 (1.59, 2.06)	<.001
Wealth index (ref: Poorest)						
Poorer	1.14 (1.06, 1.22)	<.001	1.05 (0.96, 1.16)	.276	1.04 (0.94, 1.14)	.493
Middle	1.39 (1.29, 1.50)	<.001	1.08 (0.99, 1.19)	.091	1.11 (1.01, 1.23)	.039
Richer	1.53 (1.41, 1.67)	<.001	1.13 (1.03, 1.25)	.012	1.25 (1.12, 1.40)	<.001
Richest	1.58 (1.42, 1.77)	<.001	1.20 (1.07, 1.34)	.002	1.80 (1.58, 2.05)	<.001
Area of residence (ref: Rural)						
Urban	1.20 (1.11, 1.30)	<.001	1.11 (1.03, 1.19)	.008	1.18 (1.09, 1.29)	<.001
Division (ref: Dhaka)						
Barishal	0.69 (0.62, 0.77)	<.001	1.28 (1.14, 1.44)	<.001	0.98 (0.86, 1.12)	.794
Chattogram	0.82 (0.75, 0.89)	<.001	1.01 (0.93, 1.1)	.745	0.80 (0.73, 0.89)	<.001
Khulna	2.07 (1.91, 2.26)	<.001	1.75 (1.6, 1.90)	<.001	0.61 (0.55, 0.67)	<.001
Mymensingh	1.73 (1.51, 1.98)	<.001	1.56 (1.38, 1.76)	<.001	0.70 (0.62, 0.80)	<.001
Rajshahi	1.38 (1.25, 1.51)	<.001	1.09 (0.99, 1.20)	.074	1.13 (1.01, 1.26)	.040
Rangpur	1.15 (1.04, 1.27)	<.001	1.99 (1.78, 2.22)	<.001	0.84 (0.74, 0.94)	.003
Sylhet	2.27 (2.03, 2.54)	<.001	6.38 (5.56, 7.32)	<.001	1.59 (1.40, 1.81)	<.001
Age of house head (15-34 years)						
35-50	1.21 (1.13, 1.29)	<.001	0.96 (0.90, 1.03)	.293	1.05 (0.97, 1.14)	.195
>50	1.10 (1.03, 1.18)	.007	1.00 (0.92, 1.07)	.921	0.99 (0.92, 1.08)	.865
Gender of house head (ref: Male)						
Female	1.06 (0.98, 1.15)	.143	0.92 (0.84, 1.01)	.065	1.00 (0.91, 1.09)	.922
Education of house head (ref: None or pre-primary)						
Primary	1.17 (1.11, 1.24)	<.001	0.97 (0.91, 1.05)	.456	0.91 (0.84, 0.98)	.015
Secondary	1.28 (1.20, 1.37)	<.001	1.04 (0.97, 1.12)	.264	1.01 (0.93, 1.10)	.790
Higher secondary	1.79 (1.60, 2.01)	<.001	1.14 (1.04, 1.26)	.006	1.11 (1.00, 1.23)	.057
Media exposure (ref: No)						
Yes	1.79 (1.70, 1.89)	<.001	1.18 (1.10, 1.26)	<.001	1.19 (1.11, 1.27)	<.001
Mobile phone ownership (ref: No)						
Yes	1.43 (1.36, 1.51)	<.001	1.29 (1.21, 1.37)	<.001	1.12 (1.05, 1.21)	.001

Abbreviation: HIV/AIDS, human immunodeficiency virus/acquired immune deficiency syndrome.

Women aged under 26 years of age with higher level of education, from well-off families and living in urban areas were more likely to have higher knowledge about HIV and have had a better understanding about the misconceptions of HIV. Similarly, women lived under educated house head aged below 34 years were highly likely to have had higher knowledge and awareness about HIV/AIDS transmission. The association between level of education and knowledge about HIV/AIDS, knowledge about HIV/AIDS transmission and knowledge on misconceptions about transmission of HIV/AIDS were found to be significantly associated with levels of education. Women with primary, secondary and higher secondary or above education were 2.00, 6.43 and 43.13 times more likely (*P* < .001) to have had heard about HIV/AIDS respectively, 1.18, 1.79 and 3.35 times more likely to have knowledge on HIV transmission respectively, and 12%, 28% and 81% more likely to have had knowledge about misconceptions of HIV respectively compared to their illiterate counterpart (Table 3).

Residents from Sylhet and Dhaka seem to be better equipped with different levels of HIV/AIDS knowledge. Women’s living in Barisal and Chattagram divisions had 31% and 18% significantly less knowledge about HIV/AIDS than the women from Dhaka division. However, women living in Khulna, Sylhet, Mymensingh, Rajshahi and Rangpur divisions had significantly higher level of knowledge about HIV/AIDS than the women living in Dhaka division. The detailed spatial variations of overall knowledge about HIV/AIDS among the women in Bangladesh were presented in the district-wise maps (Figure 1).

## Discussion

 In this study, a nationally representative pooled data form the latest sixth round of the MICS conducted in 2019 were analysed to estimate a district-wise proportion regarding knowledge on HIV/AIDS, knowledge on HIV/AIDS transmission and knowledge on misconceptions of HIV/AIDS of women in Bangladesh; assess its association with the sociodemographic risk factors; and identify the most vulnerable cohorts through a spatial analysis. The results highlighted that only 60.3% of Bangladeshi women have ever heard of HIV/AIDS. Furthermore, the data revealed that, amongst those women who heard of HIV/AIDS, the majority (71.7%) had knowledge about misconception of HIV/AIDS and more than half (52.2%) had good knowledge about HIV/AIDS transmission. It was found that not having access to media, not owning a mobile phone, living under an illiterate house head, residing in rural areas, belonging to the poorest quintile of wealth index and having low level of educational were significantly associated with greater likelihood of having lower level of knowledge and lower level of knowledge about HIV/AIDS, its transmission and increased misconception about HIV/AIDS, which are consistent with past literature.

Despite the current low prevalence rate of HIV/AIDS, the risk of further expansion of HIV remains high in Bangladesh due to the associated risk factors.^7,9,10,35^ Special policy attention targeting specific intervention programs will be required to make proper use of this opportunity to control further spread of the deadly virus in the country. The findings of this study suggest that overall average knowledge about HIV/AIDS and knowledge on its transmission among the women in Bangladesh is relatively low. These findings are consistent with previous research conducted in the South East Asian region and around the globe.^17,18,23^

The significant association between the overall knowledge about HIV/AIDS and exposure to media and ownership of mobile phone among the women in Bangladesh were observed in the spatial graphs (Figures 1-2). This study revealed that women in Bangladesh with no exposure to media and not having ownership of a mobile phone were less likely to have adequate knowledge about HIV/AIDS, about HIV/AIDS transmission and misconceptions about HIV/AIDS compared to their counterparts. Past studies demonstrated that media campaigns are an effective awareness dissemination tool to increase the knowledge and awareness about HIV/AIDS for the women living in rural and remote areas where literacy rate is relatively low.^8,36,37^

Healthcare promotions have proven to be effective despite adjusting for socioeconomic backgrounds in increasing awareness and greater access to health services in Bangladesh, which could be a cost-effective process to control the HIV/AIDS infection rate in Bangladesh.^19^ Past studies showed that dedicated television shows^38^ and electronic media exposure^18^ could be effective as HIV prevention campaigns. For Bangladesh government AIDS policy to have adequate impact in the community, there is a need for the government and the non-governmental organizations to work together to design and implement effective community based interventions such as awareness campaigns.^39^ The results from the current study indicated that efficient application of the media and mobile phones could help in increasing nationwide HIV/AIDS awareness.

Results revealed that better-educated and wealthier women in urban areas could more easily identify myths and misconceptions surrounding transmission. Consistent with past literature, it is expected that women with institutional education would understand the concept of how a virus spreads, comprehend the deadliness of STDs and so render awareness.^23,34^ Family solvency allows women to afford STD preventive measures, avoid the risky blood transfusions or unsafe drug habits. Residing in urban areas lead to higher exposure to information, tend to have better civil rights and have access to services provided by non-governmental organizations, which magnify awareness on STDs including HIV/AIDS.

The spatial mapping showed that women from capital city Dhaka were more likely to possess knowledge about misconception of HIV when compared with women from most of the other divisions of the country. The districts neighbouring divisional cities also seemed to have had higher media access and more likely to own personal mobile phones.^23,34,40,41^ Ownership of mobile phone lead to direct access to government information on public health campaigns, for example, Bangladesh government regularly sends out information on vaccination program through text messages.^19,42,43^ Mobile phone ownership also increases communication beyond close neighbours and increases the scope of m-health, which in turn increases citizen knowledge on diseases and their prevention.^44^

The young generation are more likely to be acquainted with social media as they regularly use mobile phones and follow mass media stories, which help them to be aware about STDs. Also, social media campaigns target youths and provide some form of sex education,^45^ which is not officially part of school curriculum in Bangladesh. This study found that younger women (≤25 years) were more likely to have knowledge about HIV/AIDS compared to older women (>25 years), which was consistent with literature.^8,46,47^ HIV/AIDS awareness campaigns and policy interventions should target social media through advertises and could build youth friendly sex education-based mobile applications.

Education of house head and their age were found to have impact on women’s overall knowledge about HIV/AIDS. Women living with house heads having primary or higher level of education and younger (<34 years) showed significantly higher level of knowledge about HIV/AIDS. These findings are corroborated with the findings of the previous study where education was identified as a ‘social vaccine’ against HIV/AIDS.^48^ Investment should also be made to aware older horseheads, so that knowledge dissemination can capture all sections of the community. Findings of this study revealed that to prevent HIV/AIDS infection knowledge and awareness about HIV/AIDS need to be increased among the women and her neighbouring factors in Bangladesh, which supports the adopted theoretical framework of the HMB. Contrary to the expectation, gender of the house head was not significantly associated with knowledge about HIV/AIDS, about HIV/AIDS transmission and misconceptions about HIV/AIDS. This could be due to the development of women’s empowerment occurred during last decade through their participation in the labor market.^49,50^ Previous studies demonstrated that women from male-headed households having lower HIV/AIDS knowledge.^46^ Previous studies have also raised the importance of gender inequality as factors to be incorporated in prevention strategies for HIV/AIDS.^51^

 To the best of our knowledge, this is the first study based on the most recent MICS (2019), a relatively large dataset representative to the population, to evaluate the impact of vital rick factors of HIV/AIDS knowledge and awareness in Bangladesh. However, this study had some limitations. First, the MICS data on HIV/AIDS was based on self-reported information received from respondents, suggesting that the data may be subject to recall bias. Second, the responses on knowledge about HIV/AIDS and its transmission and misconceptions were dichotomous, which might not be appropriate in all cases as some could have confusion regarding their responses on myths and misconceptions. Third, and knowledge on HIV was evaluated by only question as opposed to misconceptions and transmissions. Fourth, given that the MICS surveys were cross-sectional, causal associations cannot be inferred. Finally, although education of women was assessed, health literacy could not be addressed due to the limitation in the survey data, which might be more relevant for understanding health awareness and future studies/surveys could consider addressing this issue.

## Conclusion

 This study examined the most recent status of overall knowledge on HIV/AIDS, knowledge on its transmission and knowledge of misconceptions about HIV/AIDS, and their determinants among the women in Bangladesh using the MICS 2019. The findings indicated that there were multifaceted scopes for improvement in increasing knowledge about HIV/AIDS and clarifying misconceptions regarding the mode of transmission of HIV/AIDS. Exposure to media, ownership of mobile phone, education, area of residence and wealth condition were significantly associated with the knowledge of HIV/AIDS among the women in Bangladesh which reiterates the findings of previous studies that these factors need to be incorporated in the national level HIV/AIDS prevention strategies. Overall knowledge and positive attitudes are keystones of HIV/AIDS prevention, control of transmission and treatment, this study supports the significance of women’s education and exposure to mass media, and to address the uneven impact of HIV/AIDS on women’s life.

 Findings of this study suggest that mass media should take greater responsibility in HIV/AIDS related health education, especially people living in the remote and rural areas. In this regard, the radio and TV can play an important role in increasing knowledge and awareness regarding STDs among women as well as prevention of HIV/AIDS. Strengthening educational programs could have great importance in fostering knowledge and awareness among the younger generation. Furthermore, health promotion programs through community involvement could yield success in Bangladesh, where majority of the people are conservative religious minded, and women’s autonomy is limited.

## Ethical issues

Ethics approval for this study was not required since the data is secondary and available in the public domain. MICS raw data are stored without personally-identifiable information and the survey data used in this study are publicly available (https://mics.unicef.org/surveys). Before accessing the data, permission was taken from the MICS program authority by the authors.

## Competing interests

 Authors declare that they have no competing interests.

## Authors’ contributions

 JB conceptualized the study, conducted literature review, and drafted the manuscript. RKB structured the hypothesis, conducted the analysis, coded the maps, and critically reviewed the manuscript. The final manuscript was read and approved by all the authors.

## Funding

 This research received no specific grant from any funding agency in the public, commercial, or not-for-profit sectors.
